# Effect of hempseed meal on health, growth performance, ruminal fermentation, and carcass traits of intact male goats

**DOI:** 10.1093/tas/txae181

**Published:** 2024-12-25

**Authors:** Khim B Ale, Frank W Abrahamsen, Arthur L Goetsch, Jason T Sawyer, Olga Bolden-Tiller, Chukwuemeka Okere, Reshma Gurung, Santosh Chaudhary, Nar K Gurung

**Affiliations:** Department of Agricultural and Environmental Sciences, College of Agriculture, Environment and Nutrition Sciences, Tuskegee University, Tuskegee, AL 36088, USA; Department of Agricultural and Environmental Sciences, College of Agriculture, Environment and Nutrition Sciences, Tuskegee University, Tuskegee, AL 36088, USA; School of Agriculture and Applied Sciences, Langston University, Langston, OK 73050, USA; Department of Animal Sciences, College of Agriculture, Auburn University, Auburn, AL 36849, USA; Department of Agricultural and Environmental Sciences, College of Agriculture, Environment and Nutrition Sciences, Tuskegee University, Tuskegee, AL 36088, USA; Department of Agricultural and Environmental Sciences, College of Agriculture, Environment and Nutrition Sciences, Tuskegee University, Tuskegee, AL 36088, USA; Department of Agricultural and Environmental Sciences, College of Agriculture, Environment and Nutrition Sciences, Tuskegee University, Tuskegee, AL 36088, USA; Department of Agricultural and Environmental Sciences, College of Agriculture, Environment and Nutrition Sciences, Tuskegee University, Tuskegee, AL 36088, USA; Department of Agricultural and Environmental Sciences, College of Agriculture, Environment and Nutrition Sciences, Tuskegee University, Tuskegee, AL 36088, USA

**Keywords:** carcass characteristics, feed, goat, hempseed meal, ruminal fermentation, serum metabolites

## Abstract

Hempseed meal (HSM) is a potential alternative feedstuff for livestock due to its high protein content, but it has not been approved for animal feed in the United States due to safety concerns. This study was conducted to determine the effects of HSM on feed intake, growth performance, serum biochemistry, ruminal papillae morphology, ruminal fermentation profiles, and carcass characteristics of intact male goats. Thirty-six Boer × Spanish intact male goats were randomly assigned to one of four experimental diets (*n* = 9 goats/diet): 0%, 10%, 20%, and 30% HSM on as-fed basis. Diets were formulated to be isonitrogenous and fed free-choice for ad libitum consumption in the 60-day experiment. Linear and quadratic effects of different concentrations of HSM were determined. Total feed intake, crude protein intake, ruminal papillae morphology (papillae density, length, width, absorptive surface area, stratum corneum, and stratum granulosum thickness), carcass traits (longissimus muscle area, body wall thickness, backfat thickness, shrink percentage), and serum concentrations of most metabolites were similar among treatments (*P* > 0.05). Intake of acid detergent fiber (ADF) and nitrogen detergent fiber (NDF) increased linearly (*P* < 0.05) with increasing inclusion of HSM in the diet. Average daily gain (ADG), gain-to-feed ratio, and dressing percentage decreased linearly (*P* < 0.05) with an increasing percentage of HSM in the diet. Similarly, concentrations of propionic, butyric, iso-butyric, valeric, and isovaleric acids in ruminal fluid decreased linearly (*P* < 0.05) with an increasing percentage of HSM in diet, whereas concentration of acetic acid and total volatile fatty acids in ruminal fluid tended to decrease linearly (*P*-value nearly 0.10) with an increasing percentage of HSM in diet. The level of blood urea nitrogen increased linearly (*P* < 0.05) with increasing concentration of HSM, but values were within the normal range for goats. Findings from the serum metabolite analysis and ruminal papillae morphometrics suggest that goats can be fed HSM at a concentration of up to 30% in their diet. However, growth performance results indicate that further cost-benefit analysis is required to compare HSM with other commonly used protein sources such as soybean meal. These findings will be useful for legal bodies to review during the approval process of HSM as a feed ingredient for goats in the United States.

## Teaser

Food and Drug Administration-Center of Veterinary Medicine requires data on the effect of hempseed meal on each animal species and in each of their life stages before approval of hempseed meal (a potential alternative protein source) as an animal feed ingredient. Outcomes of this study will be helpful for the review during the approval process of hempseed meal as a feed ingredient for goats.

## INTRODUCTION

Hemp is defined by the 2018 Farm Bill as *Cannabis sativa* L. plant, or derivatives thereof, that contain less than 0.3% delta-9 tetrahydrocannabinol (THC) on a dry weight basis (Congress, 2018). Hemp has been cultivated for centuries worldwide for fiber and oilseed which are utilized to manufacture several products (Johnson, 2019). The United States was a large producer of hemp until the Marijuana Tax Act of 1937, which placed all Cannabis cultures under the regulatory control of the U.S. Treasury Department due to the fear of Cannabis with intoxicating concentrations of delta-9 THC being used as marijuana (Deitch, 2003). After the 2018 Farm Bill, hemp has been defined and industrial hemp has been legalized to be grown due to several beneficial properties. In the United States, the hemp industry has been focusing more on the production of oilseed and cannabidiol (CBD), which is considered to have substantial medical potential (Cherney and Small, 2016). When the hempseed is pressed, and oil is extracted, some residues remain, namely the byproduct hempseed meal (HSM). HSM contains a high amount of protein ranging from 30% to 35% crude protein (CP) and 11% to 13% fat (Mustafa et al., 1999; Hessle et al., 2008; Bailoni et al., 2021).

The demand for goat meat is increasing in the United States. In 2021, the United States imported 18,985 metric tons of goat meat worth $140 million, which is 268.6 times more than imported in 2008 (FAOSTAT, 2022). Reasons behind the increase in the demand for goat meat include an increase in people of ethnic groups who prefer goat meat, increasing popularity of goat meat among the people of the United States, and its beneficial nutritional value. However, the total population of goats in the United States on January 1, 2023, was estimated to be 2.51 million, which is 19.6% less than the goat population in 2008 (United States Department of Agriculture-National Agricultural Statistics Service [USDA NASS], 2023). The U.S. goat industry has been facing several challenges, including increased production costs, inadequate marketing systems for goats, insufficient proximity of goat meat processing facilities to areas with high production levels, and the problem of internal parasites (Gillespiex et al., 2013). As 60% to 70% of expenses in livestock production are for feed, reducing the feed cost is essential for goat producers to make significant profit. Thus, alternative feedstuffs for reducing the feed cost are important for viable goat production.

The high concentration of protein in HSM makes it a possible alternative feedstuff for use in livestock production systems. However, due to the safety concerns over the health of animals and consumers of animal-derived products, HSM has not been approved for use in animal feed in the United States by the Food and Drug Administration-Center for Veterinary Medicine (FDA-CVM). Before approval of HSM as an animal feedstuff, the FDA-CVM is required to review scientific data regarding the effects of HSM in every animal species and at every stage of their life. The American Association of Feed Control Officials (AAFCO) has also been requesting data from researchers and industry for review. Additionally, the FDA-CVM has requested that only data from studies using American grown and American processed hemp be submitted. Because there are no data available regarding the effect of HSM in diets of intact male goats for review, this research was conducted with the following objectives:

1. Feed intake and growth performance of intact male goats
2. Ruminal fermentation and ruminal papillae morphology of intact male goats
3. Serum biochemistry profile and carcass characteristics of intact male goats

## METHODOLOGY

### Experiment Site and Animal Care and Handling

The experiment was conducted at the Caprine Research and Education Unit at the George Washington Carver Agricultural Experiment Station, Tuskegee University, Alabama, United States. All experimental procedures, care, and handling of animals were approved by the Institutional Animal Care and Use Committee of Tuskegee University (Protocol Request Number R07-2019-5). This study was not conducted under the Investigational New Animal Drug guidance.

### Experimental Design and Animals

Thirty-six Boer × Spanish intact male goats (*Capra hircus*) 10 to 11 months of age were purchased and transported from one vendor in Texas. Upon arrival, animals were inspected by a veterinarian and dewormed with Cydectin (Moxidectin @1 mg/kg body weight; Bayer Health Care LLC). Animals were quarantined for 30 days and then moved to an indoor building where they were individually housed in 1.1 m × 1.2 m pens with plastic-coated expanded metal slatted floor. Goats, with a body weight of 44 ± 3.4 kg, were randomly divided into four groups (*n* = 9/group) and assigned to one of the four experimental diets using a completely randomized design.

### Experimental Diets and Compositions

Four different experimental diets with 0%, 10%, 20%, and 30% HSM on an as-fed basis were formulated as total mixed rations (TMRs). The concentration of ingredients other than HSM were adjusted based on nutrient value on book to formulate the experimental diets as isonitrogenous and to meet or exceed the nutrient requirements of growing goat as per requirements of National Research Council (NRC, 2007). The composition of each diet is presented in Table 1. Soybean meal (solvent extracted, 44% CP) and alfa-alfa meal in treatment diets was increasingly replaced by the HSM as HSM is rich in protein. Proportion of cottonseed hulls was also adjusted to make diets isonitrogenous. The HSM used was prepared by the cold press method of extracting hempseed oil. The process involved crushing hemp into a pulpy mash using super-cooled machines to ensure it remained heat-free. Pressure was applied to extract oils, resulting in organic cold-pressed CBD oil from Kentucky Hemp Works (Crofton, KY).

**Table 1.** Composition of experimental diets used in a 60-day feeding period (as-fed basis)

Feedstuff^1^	Dietary concentration of HSM supplementation, %			
	0	10	20	30
HSM, %	0	10	20	30
Cottonseed hulls, %	20	20	20	23.2
Alfalfa meal, %	30	25	25	16
Cracked corn, %	30.6	30.9	27.2	27.3
Soybean meal, %	15.9	10.6	4.3	0
Cane molasses, %	2.5	2.5	2.5	2.5
Premix^2^, %	1	1	1	1

^1^Values are presented in as-fed basis.

^2^Purina Goat Mineral with a minimum of 15.3% calcium and 8% phosphorous, 50 mg/kg selenium, 4,000 mg/kg zinc, 660,793 IU/kg. Vitamin A.

An adjustment period of 2 weeks was provided for adaptation to the indoor barn feeding system and diet acclimatization before data were collected. After acclimation, the diets were fed free-choice for ad libitum intake along with a continuous supply of drinking water via a nipple delivery system. The initial level of feeding was 3% of body weight (BW), thereafter adjusted every 3 days according to the amount of feed refusal. Feed offered and refused was weighed and recorded daily. If the amount of refusal for the previous 3 days was less than 10% of that offered, then the amount was increased by 100 g for the next 3 days. Diets were offered twice daily at 0600 and 1600 hours throughout the 60-day experiment.

### Sample Collection of Feed and Analysis

From every fifth bag of feed opened (22.68 kg), a 230-g sample was collected and composited by treatment diet at the end of experiment. Four composite samples (one composite sample per treatment diet) were sent to Holmes Laboratory (Millersburg, OH) for nutrient analysis. Nutrient analyses were conducted according to methods described by the Association of Official Analytical Chemists (AOAC, 1996): dry matter (DM) by Karl fischer titration methods (method 2001.12), CP by Kjeldahl method (method 954.01), ash (method 942.05), and ether extract (method 920.39). Calcium and all other minerals were measured by atomic absorption spectrophotometric method (method 968.08, AOAC, 1996), and phosphorous was measured by photometric method (method 965.17, AOAC, 1996). Neutral detergent fiber (NDF) and acid detergent fiber (ADF) were measured following the procedures outlined by Van Soest et al. (1991) using an Ankom^200^ Fiber Analyzer (Ankom Technology, Macedon, NY). Lignin content was assessed by incubating ADF residues in diluted sulfuric acid (1,634 g/L at 20 °C) for 3 hours in a Daisy Incubator (ANKOM Technology Corp.).

### Growth Performance Parameters

Body weight was determined on day 0 and 60 of the trial before feeding in the morning using the Sheep and Goat Weigh Scale of Lakeland Farm and Ranch Direct (Waterford, MI) to calculate live weight gain (LWG), average daily gain (ADG), and gain-to-feed ratio.

### Serum Biochemistry Profile

For analysis of serum metabolites, blood samples were collected at the end of the trial on day 60. Ten milliliters of blood were collected via the jugular venipuncture into a collection tube with clot activator (Becton Dickinson Vacutainer Serum Blood Collection Tubes). Then, blood samples stored on ice were sent to Veterinary Diagnostic Laboratory, Tuskegee University, Tuskegee, Alabama, on the same day for determination of the serum biochemistry profile with the IDEX Catalyst DX system (IDEXX Laboratories, Inc., Westbrook, ME).

### Ruminal Fermentation and Ruminal Papillae Morphology

Animals were processed on day 60 at the Lambert-Powell Meats Laboratory, Auburn University, Alabama, following USDA standards. To analyze volatile fatty acid concentrations, a ruminal fluid sample (15 mL from each animal) was collected in 50 mL test tubes within 10 minutes after the slaughter, and 3 mL of 6 *N* HCL was added to the sample. Then, samples were frozen at 0 °C for at least 24 hours and transported to the Cumberland Valley Analytical Services (CVAS, Bellefonte, PA) on dry ice. At CVAS, 3 mL of centrifuged extract was filtered through a 0.22-micron filter membrane. An internal standard was added, and a 0.5 µL subsample was injected into a Perkin Elmer AutoSystem Gas Chromatograph using a Nukol capillary column (Supelco, Bellefonte, PA), and concentrations of acetic, propionic, butyric, iso-butyric, valeric acid, and isovaleric acids were analyzed.

For the histomorphology study, a 1 cm × 1 cm rumen tissue sample was collected from the ventral sac of the rumen from each animal, and samples were cleaned with water and preserved in formalin (10% formaldehyde) until further processing. Morphometric variables of density (counts per cm^2^), and length and width of papillae were studied. For density, the number of papillae present in the tissue sample was counted manually, and the length and width of five ruminal papillae were measured using a vernier caliper (Spurtar Vernier Caliper, Kings Company, China), with calculation of an average value. To estimate the absorptive surface area of ruminal papillae, the following equation was used:


$$\begin{array}{lll} Absorptive\;Surface\;Area=\; & 2*Length\;of\;papillae*Width\;of\;Papillae\; & *Papillae\;density \end{array}$$

**Figure 1.**
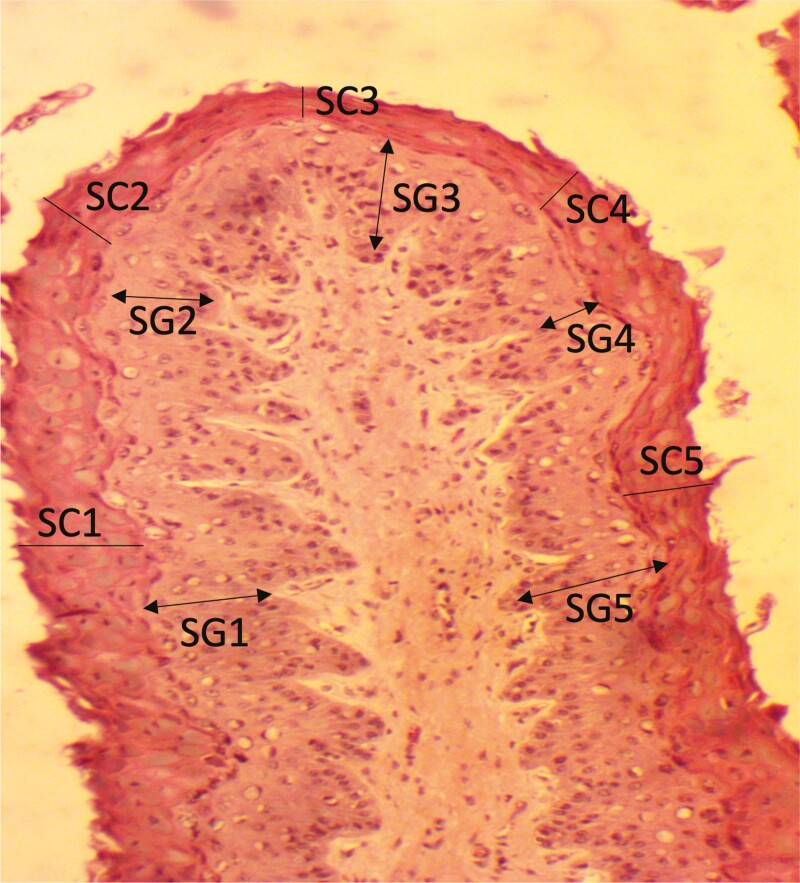
Histology slide of ruminal papillae showing stratum corneum (SC) and stratum granulosum (SG) thickness measured at five different points.

To study the effect on stratum corneum thickness and stratum granulosum thickness of the ruminal papillae, histological slides of rumen tissue samples were prepared using hematoxylin and eosin stain. The thickness of the stratum corneum and stratum granulosum (Figure 1) was measured at five points in the histological slide under the microscope using a camera (i.e., AmScope FMA050 Fixed Microscope Adapter).

### Carcass Characteristics

Feed and water were withheld overnight before slaughter. Live body weight of animals was measured before slaughter at the Lambert-Powell Meats Laboratory, Auburn University, Alabama. Animals were slaughtered following the guidelines by the United States Department of Agriculture-Food Safety and Inspection Service (USDA FSIS), which comply with the Humane Method of Slaughter Act (USDA FSIS, 2015). After slaughter, the head, skin, intestinal tract, and internal organs were removed. Then, the carcass was rinsed using hot water and a 2% solution of lactic acid, prior to recording hot carcass weight (HCW) using a Static Monorail Scale (Vandenberg Scales, Sioux Center, IA). Carcasses were chilled at 1 °C for 24 hours, and they were re-weighed using the Static Monorail Scale to record the cold carcass weight (CCW). Longissimus muscle area (LMA) was determined by measuring the surface area of the longissimus dorsi muscle between the 12th and 13th ribs of the carcass using a grid, with the grid method being accurate, feasible, and practical (Yáñez et al., 2006). Subcutaneous fat over the REA (at the point 3/4th the length of ribeye from split chine bone) was measured using a caliper, and the body wall thickness was measured in the 12th rib approximately 11.43 cm from the midline of the goat carcass.

As HSM is not yet approved as a feed ingredient for livestock in the United States, the meat produced in this study was not eligible for human consumption or market distribution. Therefore, following the completion of the carcass characteristics analysis, the carcasses were disposed of via incineration.

### Statistical Analysis

Data were entered into an Excel spreadsheet and processed with the R version 4.2.1. Orthogonal contrast tests for equally spaced treatment were employed to examine the linear and quadratic effect of different dietary concentrations of HSM. The significance threshold used was *P* <0.05, and a *P*-value between 0.05 and 0.10 indicates a trend or tendency.

## RESULTS

### Feed Composition

The nutrient composition of the diets and HSM is shown in Table 2. HSM used in this experiment was high in CP (36.4%), total digestible nutrients (TDNs) (63.2%), and fat (11.5%). Slight numerical differences in concentration of CP in the experimental diets were observed. Concentration of ADF, NDF, lignin, and fat increased in the diet with the increase in percentage of HSM. TDN decreased with increasing HSM inclusion, with 0% HSM diet having 69.72% TDN and the 30% HSM diet containing 65.05%. The net energy for lactation (NEl), net energy for maintenance (NEm), and net energy for growth (NEg) followed a similar trend, with the values decreasing as HSM concentration in the diet was increased.

**Table 2.** Analyzed nutrient composition of experimental diets fed to Boer × Spanish intact male goats for a 60-day feeding period and nutrient composition of HSM used for formulation of experimental diets

Nutrient analysis^1^	Dietary concentration of HSM, %				HSM
	0	10	20	30	
Moisture, %	10.26	10.71	9.98	10.67	11.4
DM, %	89.74	89.29	90.02	89.33	89.6
CP, %	17.55	17.68	18.25	17.33	36.42
Available protein, %	16.81	16.91	17.29	16.32	—
ADF protein, %	0.74	0.77	0.96	1.01	—
NDF protein, %	3.28	3.6	4.05	4.32	—
Lignin, %	5.64	5.9	8.21	9.07	12.26
ADF, %	24.45	25.49	31.78	33.32	36.47
NDF, %	32.6	35.74	40.27	42.98	49.47
NFC, %	42.1	39.21	34.06	32.84	10.47
Crude fat, %	3.45	3.82	4.43	4.8	11.53
TDN, %	69.72	69.28	65.87	65.05	63.22
NEl, MJ/kg	6.64	6.64	6.27	6.18	—
NEm, MJ/kg	6.82	6.73	6.27	6.18	—
NEg, MJ/kg	4.24	4.24	3.78	3.69	0.38
Ash, %	6.72	6.35	6.34	5.7	5.82
Lignin insoluble, %	0.21	0.21	0.21	0.21	—
Calcium, %	0.74	0.64	0.61	0.55	0.23
Phosphorus, %	0.35	0.41	0.45	0.51	1.03
Magnesium, %	0.22	0.23	0.25	0.28	0.49
Potassium, %	1.49	1.37	1.33	1.09	1.03
Sulfur, %	0.2	0.22	0.23	0.24	0.16
Sodium, %	0.17	0.11	0.13	0.11	0.03
Copper, mg/kg	161	154	169	158	37
Manganese, mg/kg	453	433	494	476	88
Zinc, mg/kg	555	553	598	521	76
Iron, mg/kg	142	148	159	132	120
Nitrate	Negative	Negative	Negative	Negative	Negative

^1^All values are on a DM basis except for the moisture and DM, and energy values were calculated as described by Weiss (1993).

Available protein = CP − ADF protein.

Metabolizable energy (ME) = 0.82* TDN* 0.04409.

NEl (MCAL/LB) = (TDN * 0.0111) − 0.054.

NEm MCAL/LB = (11.37 * МЕ − 0.138 * МЕ^2 + 0.0105 * МЕ^3 − 1.12) * 0.4535.

NEg MCAL/LB = (1.42* ME − 0.174 * ME*2 + 0.0122 * ME^3 − 1.65) * 0.4535.

1 MCAL/LB = 9.30 MJ/kg.

### Dry Matter Intake, Growth Performance, and Feed Efficiency

There were no significant differences (*P* > 0.05) in intake of DM or CP. However, intake of NDF and ADF increased linearly (*P* < 0.05) as the dietary concentration of HSM increased. As the proportion of HSM in the diet increased, LWG, ADG, and gain-to-feed ratio decreased linearly (*P* < 0.05). LWG was 22.1, 20.8, 18.7, and 18.5 kg in the 60-day trial period, ADG was 369, 346, 311, and 308 g/day, and gain-to-feed ratio was 0.14, 0.14, 0.12, and 0.12 for goats fed diets with 0%, 10%, 20%, and 30% HSM, respectively.

### Ruminal Fermentation and Ruminal Papillae Morphology

Volatile fatty acid concentrations in ruminal fluid are presented in Table 3. There was no significant difference (*P* > 0.05) in the acetic acid concentration among diets with 0%, 10%, 20%, and 30% HSM. However, acetic acid concentrations tended to decrease linearly (*P* = 0.1) with increasing inclusion of HSM. Propionic, butyric, iso-butyric, valeric acid, and isovaleric acid concentrations decreased linearly (*P* < 0.05) as the concentration of HSM in the diet increased. Similarly, the acetic acid-to-propionic acid (A:P) ratio increased linearly (*P* < 0.05) with increasing concentration of HSM in the diet, whereas the total volatile fatty acid concentration also tended to decrease linearly with increasing concentration of HSM in the diet (*P* = 0.06).

**Table 3.** Volatile fatty acid concentrations in ruminal fluid and ruminal papillae morphometrics of Boer × Spanish intact male goats fed diets with varying levels of HSM for 60 days

Item	Dietary concentration of HSM^1^, %				SEM	*P*-value^2^	
	0	10	20	30		Linear	Quadratic
Volatile fatty acids							
Acetic acid, mM	64.8	56.3	43.2	47.7	8.54	0.10	0.45
Propionic acid, mM	12.9	9.5	8.1	7.3	1.54	0.04	0.40
Butyric acid, mM	4.50	2.68	2.84	2.80	0.399	0.01	0.03
Iso-butyric acid, mM	0.90	0.58	0.57	0.62	0.058	0.002	0.002
Valeric acid, mM	0.91	0.59	0.49	0.54	0.083	0.002	0.035
Isovaleric acid, mM	1.69	0.97	1.03	1.15	0.156	0.033	0.012
A:P	5.07	5.90	5.55	6.62	0.339	0.002	0.035
Total volatile fatty acids, mM	85.7	70.6	56.2	60.2	10.5	0.06	0.37
Ruminal papillae morphometrics							
Papillae density, counts/cm^2^	42.1	42.9	52.3	49.9	4.19	0.24	0.09
Length, mm	6.03	7.50	6.69	6.82	0.48	0.47	0.17
Width, mm	2.77	2.25	2.67	2.63	0.197	0.992	0.226
Absorptive surface area, mm^2^	1428	1487	1836	1735	203	0.17	0.70
Stratum corneum, µm	9.21	9.58	10.64	10.05	0.885	0.371	0.593
Stratum granulosum, µm	23.8	23.6	22.2	22.7	1.83	0.57	0.85

A:P = acetic acid-to-propionic acid ratio.

^1^Values presented as least square mean.

^2^ *P*-value based on orthogonal contrast for equally spaced treatments (*n* = 9 per treatment).

Papillae density = number of papillae per 1 cm × 1 cm rumen tissue sample.

Absorptive surface area = 2 × length of ruminal papillae × width of ruminal papillae × papillae density.

Stratum corneum = thickness of stratum corneum measured at 5 different points/5.

The observed morphometric variables are presented in Table 3. Papillae density, width of papilla, absorptive surface area, stratum granulosum thickness, and stratum corneum thickness were not affected (*P* > 0.05) by the dietary concentration of HSM. However, the papillae density tended to follow the quadratic trend (*P* = 0.09) with increasing inclusion of HSM in the diet.

### Serum Metabolites

Serum metabolite concentrations are presented in Table 4. All the observed serum metabolites were similar (*P* > 0.05) among the treatment groups except for blood urea nitrogen (BUN). However, the BUN concentration increased linearly (*P* < 0.05) with the increasing dietary inclusion rate of HSM.

**Table 4.** Serum metabolites of Boer × Spanish intact male goats fed diets with varying concentrations of HSM for 60 days

Serum metabolites	Dietary concentrations of HSM^1^, %				SEM	*P*-values^2^	
	0	10	20	30		Linear	Quadratic
Creatinine, mg/dL	0.5	0.5	0.5	0.5	0.03	0.86	0.44
BUN, mg/dL	21.1	24.4	24.9	24.4	1.24	0.02	0.27
Glucose, mg/dL	47	50	49	48	0.78	0.35	0.67
Phosphorous, mg/dL	7.5	6.9	8.2	7.3	0.28	0.33	0.75
Calcium, mg/dL	9.1	8.9	9.1	8.8	0.16	0.23	0.66
Sodium, mmol/L	144	143	143	143	0.65	0.24	0.93
Potassium, mmol/L	6.4	6.3	6.6	6.4	0.29	0.81	0.85
Chloride, mmol/L	105	105	106	105	0.56	0.86	0.24
Bicarbonate, mmol/L	28	27	27	26	0.82	0.10	0.89
Total protein, g/dL	6.5	6.7	6.4	6.5	0.16	0.75	0.86
Albumin, g/dL	3.0	3.0	3.1	3.1	0.07	0.11	0.61
Globulin, g/dL	3.5	3.6	3.2	3.4	0.16	0.12	0.61
ALT, U/L	16.0	14.6	16.8	14.6	0.68	0.49	0.57
AST, U/L	69.3	65.7	70.8	66.8	2.67	0.83	0.95
ALP, U/L	256	235	285	299	25.4	0.13	0.51
GGT, U/L	50.6	52.1	51.98	46.4	2.75	0.31	0.21
Cholesterol, mg/dL	67.0	65.9	73.0	73.3	5.20	0.27	0.89
Amylase, U/L	30.6	33.4	27.0	30.7	1.83	0.46	0.83
Lipase, U/L	17.3	17.4	18.4	17.1	1.29	0.95	0.58
Creatine kinase, U/L	126	122	140	133	8.09	0.30	0.88

U/L = units per liter.

^1^Values presented as least square mean.

^2^ *P*-value based on orthogonal contrast for equally spaced treatments (*n* = 9 per treatment).

Serum metabolites concentrations were measured using IDEX Catalyst DX System (IDEXX Laboratories, Inc., Westbrook, ME).

### Carcass Characteristics

Carcass traits are shown in Table 5. Dressing percentage (DP) decreased linearly (*P* < 0.05) as the percentage of HSM in the diet increased, with values of 42.4%, 42.0%, 41.4%, and 40.0% for diets with 0%, 10%, 20%, and 30% HSM, respectively. Similarly, HCW and cold carcass decreased with the increasing dietary concentration of HSM. Carcass characteristics of the shrink percentage, LMA, body wall thickness, and backfat thickness were similar (*P* > 0.05) among dietary HSM concentrations.

**Table 5.** Carcass characteristics of Boer × Spanish intact male goats fed diets with varying inclusion rate of HSM for 60 days

Carcass traits	Dietary concentrations of HSM^1^, %				SEM	*P*-value^2^	
	0	10	20	30		Linear	Quadratic
DP, %	42.4	42.0	41.4	40.0	0.70	0.017	0.500
HCW, kg	28.5	27.7	26.5	25.6	0.88	0.01^**^	0.95
CCW, kg	27.9	27.2	26.0	25.0	0.82	0.01^**^	0.88
Shrink percentage, %	1.94	1.73	1.62	2.10	0.67	0.90	0.61
LMA^3^, cm^2^	34.0	32.3	35.0	33.1	1.45	1.000	0.951
Body wall thickness^4^, cm	0.81	0.81	0.68	0.58	0.107	0.092	0.619
Backfat thickness^5^, cm	0.17	0.10	0.09	0.11	0.036	0.217	0.257

SEM = standard error of mean.

^1^Values presented as least square mean.

^2^ *P*-value based on orthogonal contrast for equally spaced treatments (*n* = 9 per treatment).

$Dressing\;percentage\;=\frac{Hot\;\;\;carcass\;\;\;weight}{Live\;\;\;weight}x100\;\;\;\;\;\;\;$

$Shrink\;percentage\;=\frac{Hot\;\;\;carcass\;\;\;weight\;\;\;--\;\;\;Cold\;\;\;carcass\;\;\;weight}{Hot\;\;\;carcass\;\;\;weight}x100$

^3^Surface area of the longissimus dorsi muscle between the 12th and 13th ribs of the carcass was measured using a grid.

^4^Subcutaneous fat over the LMA measured using a caliper.

^5^Body wall thickness measured in the 12th rib approximately 11.43 cm from the midline of carcass.

## DISCUSSION

In the context of industry personnel and researchers being asked by AAFCO and FDA-CVM for scientific data about the effects of HSM in diets for animal species, including various stages of life, before approving HSM as an animal feed ingredient, there is a need for data with intact male goats. We found similarities in dry matter intake (DMI), carcass quality traits, ruminal papillae morphology, and serum metabolite concentrations among animals fed diets with varying concentrations of HSM from 0% to 30%. However, decreases were noted in growth performance, propionic acid concentration in ruminal fluid, and DP as the concentration of HSM in the diet increased.

### Nutrient Composition of Diets

HSM used in this trial was obtained from oil extraction from hempseed using the cold mechanical pressing method. The CP content of the HSM (36.4%) was similar to HSM (35%) used by Winders et al. (2022) in a study with crossbred finishing heifers. Similarly, Mustafa et al. (1999) reported a CP concentration in HSM of 32.1%, whereas in a review paper by Bailoni et al. (2021), the CP content of HSM ranged from 31.4% to 37.7%. The observed CP content of HSM was approximately 10 percentage points lower than the CP of 44% in soybean meal, which is widely used as a protein source in livestock feeds (Mavromichalis, 2018). Several authors have reported a lipid content of HSM ranging from 9.5% (Winders et al., 2022) to 17.9% (Bailoni et al., 2021). The wide range in the lipid content of HSM might be due to the difference in the efficiency of mechanical pressing to extract oil from hempseed. The NDF and ADF contents of the HSM used in this trial (49.5% and 36.5%, respectively) were similar to concentration reported by Winders et al. (2022) of 49.5% and 33.8%, respectively. However, Bailoni et al. (2021) reported an NDF concentration ranging from 33% to 37% and an ADF concentration of 21% to 23% in HSM.

In this experiment, although the diets were formulated to be isonitrogenous, nutrient analysis showed slight numerical difference in CP content among the different diets which might be due to formulation being based on nutrient value of ingredients given in the NRC (2007). Concentrations of TDN, NDF, lipid, and NEg were within the range of requirements for growing goats given by NRC (2007). However, increasing lignin, ADF, and NDF values with increasing concentration of HSM in the diet were expected because of relatively high concentration of lignin in HSM relative to soybean meal. The dietary lipid concentration also increased as the inclusion level of HSM increased because of the high concentration of lipids in the HSM used.

### DMI, CP Intake, Growth Performance, and Feed Efficiency

DMI was similar among different dietary HSM concentrations. Abrahamsen et al. (2021) also observed no difference in DMI when castrated male goats were fed TMR diets containing 0%, 10%, 20%, and 30% HSM for 60 days (Abrahamsen et al., 2021). Similarly, Antunović et al. (2020) reported that total feed intake was not affected by the inclusion of HSM in diets compared with the use of soybean meal in diets of lambs (Antunović et al., 2020). Similarly, in an experiment by Winders et al. (2021), DMI was similar between finishing heifers fed a diet with 20% distillery dried grains with soluble (DDGS) and one with 20% HSM in the diet for 111 days. CP intake was similar among the groups because diets were formulated to be isonitrogenous, and DMI was similar among treatments.

LWG, ADG, and gain-to-feed ratio decreased linearly as the dietary inclusion level of HSM increased, which might be due to decreasing TDN and NEg, and increasing concentrations of NDF, ADF, and lignin. These findings agree with those of Abrahamsen et. al. (2021) when castrated male goats were feed with diets containing 0%, 10%, 20%, and 30% HSM for 60 days (Abrahamsen et al., 2021). A similar decrease in ADG and feed efficiency was reported by Winders et al. (2021) for finishing heifers fed a diet with 20% HSM compared with a diet containing 20% DDGS. However, Smewogere et al. (2022) reported no difference in DMI, ADG, or feed efficiency when Kalahari red meat wether goats were fed diets with up to 10% of HSM for 42 days, which might be because of the low percentage of HSM used for a relatively short period of time. ADG in the present experiment of 308 to 368 g was higher than reported by previous studies. For example, Niekerk and Casey (1988) reported ADG of 291 g for male Boer kids at the age of 100 days and 250 g for male Boer goats at the age of 270 days. Abrahamsen et al. (2021) noted ADG of 179 g of Boer × Spanish wethers at 4 to 5 months of age. Semwogerere et al. (2022) found an ADG of 250 g with Kalahari goats 3 to 4 months of age. Hu et al. (2012) reported an ADG of 315 g for goats in a buck performance test in 2007. Similarly, in a meat buck performance test (84 days long) at Langston University, the mean ADG reported was 295 g, with the highest value of 408 g (Gipson, 2007) with bucks fed a diet containing 16% CP, 2.5% fat, 20.4% crude fiber, and 60.6% TDN. The observed high growth rate in this experiment could potentially be attributed to the genetic potential of Boer goats, a high plane of nutrition by feeding TMR diets with over 16% CP and 65% TDN, and individual housing of animals in pens.

### Ruminal Fermentation and Ruminal Papillae Morphology

Ruminal fermentation is the most important part of digestion in every ruminant. Morphology of the ruminal papillae changes in response to dietary characteristics. Some of the changes include but are not limited to elongation and lateral expansion, branching and proliferation of papillae, and thickening of the cornified layer of epithelium (Ferguson et al., 2022). Similarly, the concentration of volatile fatty acids also varies with substrate composition, substrate availability, passage rate, and microbial species in the rumen (Dijkstra, 1994). The decrease in molar concentration of propionic acid was expected because of the decrease in TDN and non-fiber carbohydrate (NFC) in the diet. A similar decrease in propionic acid was reported by Abrahamsen et al. (2021), with 41.3, 28.1, 17.6, and 10.1 mmol/L in ruminal fluid of castrated goats fed with 0%, 10%, 20%, and 30% of HSM diet, respectively, for 60 days. In contrast to our findings, Abrahamsen et al. (2021) also reported decreases in concentrations of other volatile fatty acids (i.e., acetic, butyric, valeric, iso-butyric, and valeric acids) along with propionic acid. In this research, the acetate concentration decreased with the increase in dietary HSM concentration. However, acetate concentration was expected to increase with the increase in HSM inclusion because of the increase in concentration of fiber in the diet with an increase in concentration of HSM (Hutjens, 1998). The optimal A:P ratio for the ruminants is more than 2.2 to 1.0 (Hutjens, 1998), and thus the observed A:P ratio of all of the experimental groups in this experiment were optimal and similar among the treatment groups.

There was no difference among diets in the total volatile fatty acid concentration in ruminal fluid, but values tended to decrease linearly (*P* = 0.06) as the concentration of HSM in the diet increased. In contrast to this research, Winders et al. (2023) found a greater concentration of total ruminal volatile fatty acids in steers fed a diet with 20% hempseed cake vs. a diet with 20% distillery dried grains with soluble (DDGS).

It does not appear that there are previous reports concerning potential effects of the dietary level of HSM on papillae morphology in any ruminant species. However, in contrast to our findings regarding the similarity in morphological characters among the experimental groups, Dirksen et al. (1984) reported that the papillary surface area was higher by a factor of 3.6 in cattle fed a diet with 32% to 37% crude fiber than in the cattle fed with 11% crude fiber diet (Dirksen et al., 1984). Additionally, Ferguson et. al. (2022) reported that SC and SG thickness was higher in beef cattle fed a diet high in cereal grain vs. a high-forage diet (i.e., 90% to 100% cereal vs 100% forage diet).

### Serum Metabolites

The serum biochemistry profile reflects the overall health of animals. In this study, all the observed serum metabolites, including alanine aminotransferase (ALT), aspartate aminotransferase (AST), alkaline phosphatase (ALP), gamma-glutamyl transferase (GGT), creatinine, and total protein, were within normal levels for goats. All the serum metabolites except BUN were not affected by the inclusion level of HSM in the diet. The BUN level increased with the increasing concentration of HSM, which was similar to findings reported by Abrahamsen et al. (2021) in castrated male goats when fed diets with HSM concentrations of 0% to 30%. An increase in dietary nitrogen intake is one of the factors responsible for an increasing BUN level in cattle (Hammond, 1983), which may also apply to goats. However, BUN level was increased with increase in HSM in diet in the present study despite having similar CP intake (Table 6) among experimental groups. House et al. (2010) reported the protein digestibility of HSM to be 90.8% to 97.5%, and Mustafa et al. (1999) reported 77.4% of total protein of HSM to be ruminal undegradable (RUP). Thus, higher RUP and higher protein degradability with the increasing concentration of HSM in the diet might have contributed for higher BUN with an increase in HSM inclusion in the diet.

**Table 6.** DMI, growth performance, and feed efficiency of Boer × Spanish intact male goats fed diets with varying concentrations of HSM for 60 days

Items	Dietary concentration of HSM^1^, %				SEM	*P*-value^2^	
	0	10	20	30		Linear	Quadratic
Initial body weight, kg	43.9	44.0	44.2	44.1	1.33	0.91	0.95
Final body weight, kg	66.1	64.7	62.8	62.6	1.79	0.13	0.76
DMI, g/day	2306	2259	2315	2217	63	0.41	0.65
ADF Intake, g/day	564	576	736	739	17.1	<0.001	0.80
NDF Intake, g/day	752	807	932	953	22.3	<0.001	0.44
CP Intake, g/day	405	399	423	384	9.82	0.40	0.11
LWG, kg	22.1	20.8	18.7	18.5	0.58	0.01	0.25
ADG, g/day	369	346	311	308	18.1	0.01	0.58
Gain: Feed ratio	0.143	0.137	0.121	0.124	0.006	0.01	0.39

SEM = standard error of mean.

^1^Values presented as least square mean.

^2^ *P*-value based on orthogonal contrast for equally spaced treatments (*n* = 9 per treatment).

### Carcass Characteristics

In similar research by Gurung et al. (2022) with castrated male goats fed diets with 0%, 10%, 20%, and 30% HSM for 60 days, LMA and body wall thickness were similar among treatments, which agrees with findings of this research. However, in the same study by Gurung et al. (2022), DP was not affected by the level of inclusion of HSM. The observed DP of the goats (i.e., 40% to 42%) was lower than reported by Gurung et al. (2022) (i.e., 45% to 46% for 6- to 7-month-old Boer × Spanish castrated goats), Semwogerere et al. (2022) (i.e., 49% to 50% for 3- to 4-month-old Kalahari wether goats), and Gurung et al. (2009) (i.e., 42% to 45% for 4- to 5-month-old Kiko × Spanish intact male goats fed with DDGS diets). However, the observed DP were comparable with DP reported by Ale et al. (2022) (i.e., 38.5% to 40.7% for 5- to 6-month-old Kiko crossbreed castrated male goats fed with 10% to 30% DDGS diet). The DP of goats is affected by various factors such as age, slaughter weight, diet, castration, sex, genotype, and gut fill (Assan, 2015). Age and higher slaughter weight as well as high growth rate might be reasons for the lower DP observed in the current study.

## CONCLUSION

Findings from the serum metabolite analysis and ruminal papillae morphology indicate that incorporating HSM into the diet of intact male goats has no adverse effects on overall health. This suggests that goats can be fed HSM in diets up to a concentration of 30%. However, growth performance, feed efficiency, volatile fatty acids in ruminal fluid, and DP were observed to decrease with the increasing level of inclusion of HSM in the diet, although values were in an acceptable range. To determine the optimal inclusion rate for profitability, further research on cost-benefit analysis is required to compare HSM to other commonly used protein sources. Furthermore, since our study used only isonitrogenous experimental diets, future research should also investigate the effects of isocaloric experimental diets. Finally, these findings will be helpful for the review by FDA-CVM and AAFCO during the approval process of HSM as a feed ingredient for goats.
